# Combined effect of exercise and curcumin on inflammation-associated microRNAs and cytokines in old male rats: A promising approach against inflammaging

**DOI:** 10.1016/j.heliyon.2025.e41895

**Published:** 2025-01-11

**Authors:** Zahra Shargh, Keyvan Asghari, Morteza Asghariehahari, Leila Chodari

**Affiliations:** a Student Research Committee, Urmia University of Medical Sciences, Urmia, Iran; b Neurophysiology Research Center, Cellular and Molecular Medicine Research Institute, Urmia University of Medical Sciences, Urmia, Iran; c Department of Physiology, School of Medicine, Urmia University of Medical Sciences, Urmia, Iran

**Keywords:** Aging, Curcumin, Exercise, Inflammation, miRNAs

## Abstract

**Purpose:**

Inflammation serves as a key contributor to various diseases, necessitating the discovery of new treatment approaches to address its causative role.

**Methods:**

The study involved 35 male Wistar rats, including 7 young rats (3-month-old; 200–250 g) in the Young control group and 28 aged rats (18-month-old; 400–450 g) randomly distributed among the Old control, Exercise, Curcumin, and Exercise + Curcumin groups. During an 8-week period, the Exercise group underwent running on the treadmill (17 m/min), while those in the Curcumin group were supplied with daily curcumin doses (50 mg/kg) through gavage. Upon completion of the study, serum samples from each group were collected for evaluating interleukin-6 (IL-6), interleukin-1β (IL-1β), interleukin-10 (IL-10), and Tumor Necrosis Factor-α (TNF-α) levels using ELISA; malondialdehyde (MDA) by enzymatic assay; and miR-21 and miR-146a by RT-PCR.

**Results:**

Our findings revealed that the Old control group, in contrast to the Young control group, showed a significant reduction in IL-10 serum levels, while MDA, TNF-α, IL-1β, and IL-6 serum levels were significantly elevated. Additionally, the expression of inflammatory microRNAs (miRNAs), miR-21 and miR-146a, was significantly enhanced in the Old control rats compared with the Young control group. Exercise and curcumin treatment alone resulted in an improvement in the expression of the markers and miRNAs associated with inflammation. Furthermore, when exercise and curcumin were administered simultaneously, a synergistic effect was observed compared to the exercise or curcumin alone groups.

**Conclusion:**

Curcumin and exercise, individually and synergistically in combination, effectively reduced inflammation in aged rats, likely due to decreased oxidative stress and MDA levels mediated by miR-21 and miR-146a downregulation.

## 1 Introduction

Aging, as a complex biological process in which a person gradually loses the ability to maintain homeostasis, is a key contributor to multiple chronic health conditions, particularly cardiovascular, renal, and hepatic diseases [1]. As the elderly population, specifically individuals who are 65 or above, is expected to grow worldwide from a 2010 estimate of 524 million to an estimated 1.5 billion in the year 2050, age-associated diseases are expected to become astonishingly more prevalent [2]. The aging process entails a state of low-level non-resolving inflammatory pathways activation, which is referred to as "inflammaging" [3].

Inflammation is a beneficial physiological response to stress situations in their acute form [4]. However, inflammation is a double-edged sword and can be harmful if not controlled properly [3]. Chronic low-level inflammation is defined as an increase in systemic concentrations of such cytokines as interleukin-6 (IL-6), interleukin-1β (IL-1β), and Tumor Necrosis Factor-α (TNF-α) [5]. Cytokines, which serve as inflammatory markers, are soluble molecules that have roles in communication between cells; and are produced by a wide range of cell types within the body, including liver cells [6]. Inflammaging is characterized by increased levels of cytokines IL-6, IL-1β, and TNF-α, all of which are proinflammatory and have been shown to rise with age and are pathogenically involved in the majority of age-associated diseases [7]. Oxidative stress is intimately associated with the aging process and has a crucial function in the development of chronic inflammation [8,9]. It happens when there is an imbalance in the equilibrium between the generation of reactive oxygen species (ROS) and the body's protective antioxidant mechanisms [10]. Increased ROS levels can lead to lipid peroxidation, leading to the creation of malondialdehyde (MDA), a marker of oxidative stress damage [11]. MDA has been shown to contribute to inflammatory pathways activation and proinflammatory cytokines release, further exacerbating the inflammatory response observed during aging [12]. IL-6 belongs to the pro-inflammatory cytokine family and stimulates the expression of numerous proteins that are accountable for acute inflammation [13]. IL-6 is unanimously recognized as an inflammatory biomarker and is frequently utilized to evaluate the existence and intensity of low-grade inflammation [14]. IL-1β is considered one of the proinflammatory cytokines, which is highly associated with inflammatory pain [15]. IL-1β, predominantly released by activated monocytes and macrophages, serves as a main inflammation driver [16]. Macrophages secrete interleukin-10 (IL-10), a major cytokine possessing anti-inflammatory and immune-suppressive attributes [17]. Michael R. Strickland, reported a correlation between IL-10 expression and diminished levels of inflammatory chemokines [18]. A decline in IL-10 levels during the aging process can contribute to age-related inflammation [19].

MicroRNAs (miRNAs) are tiny RNA molecules consisting of around 22 nucleotides that do not code for proteins, but modulate physiological as well as pathological pathways by regulating gene expression through both transcriptional and posttranscriptional mechanisms [20]. Interestingly, there are several circulating miRNAs that show potential as biomarkers for major age-related diseases marked by chronic, low-level inflammation, for instance cardiovascular disease (CVD), Alzheimer's Disease (AD), rheumatoid arthritis (RA), and various forms of cancer [21]. Recent research has shown that during aging, the number of inflammatory miRNAs gradually and regularly increases [21]. Among the inflammatory miRNAs, we can mention miR-21 and miR-146a [22], which can have a negative effect on the heart [23] and kidney [24] during aging. Physical activity, a modifiable lifestyle factor, has been linked to lower levels of inflammatory biomarkers such as IL-1β, IL-6, and TNF-α in the blood [25]. Engaging in more physical activity may be a viable strategy for reducing chronic inflammation [26]. Abd El-Kader and Al-Shreef results demonstrate that both aerobic and resistance exercise training reduce IL-6, TNF-α, and C-reactive protein (CRP) levels in elderly individuals [27].

Curcumin, the main active ingredient in turmeric, is a yellow phenolic pigment that has a wide range of biological and pharmacological activities. In addition, curcumin is a powerful antioxidant and cleanser of free radicals that can prevent the production of a variety of free oxidant radicals in the biological environment. [28] Exercise has been shown to promote improving effects on inflammation during the aging process [29]. Regular exercise has been linked to a reduction in chronic low-grade inflammation [29]. It has been observed that exercise can modulate the inflammatory response, potentially provoking a drop in the levels of proinflammatory cytokines [29]. Therefore, incorporating exercise into one's routine is deemed beneficial for reducing age-associated inflammation [29]. Based on previous studies, it is evident that both exercise and curcumin individually have the capacity to reduce inflammation. This study seeks to evaluate the impact of simultaneous administration of curcumin and exercise. Furthermore, we aim to identify the signaling pathway involved in mediating the reduction of inflammation. To achieve this, we examined miR-21 and miR-146a expressions as potential players in this signaling loop.

## 2 Materials and methods

### 2.1 Animals and study design

**Fig. 1:**
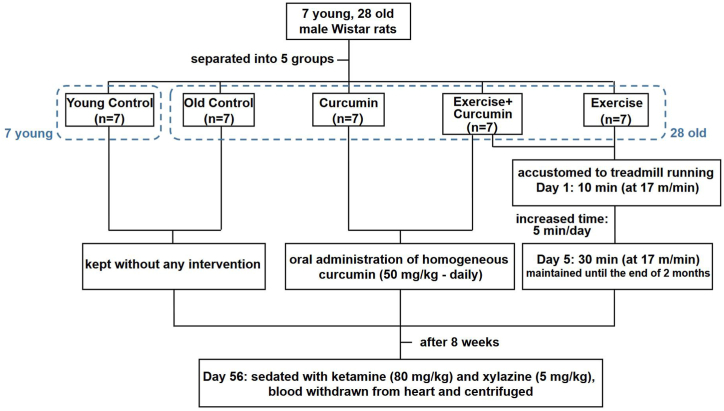
The plan and timeline of the study showing the groupings, the 8-week treatments, and the exercise training plan. The animals were divided into 5 groups (n = 7 each).

This work received approval from the Animal Ethics Committee, complying with Urmia University of Medical Sciences guidelines for the care and use of laboratory animals (IR.UMSU.REC.1399.184). The housing environment of the animals was regulated to maintain a steady temperature of 24 °C. Food and water were available ad libitum, with a 50 % relative humidity and a 12-h dark/light rhythm. There were five groups (n = 7) formed by randomly separating twenty-eight old male Wistar rats (18-month-old; 400–450 g) to four groups; and one group of young rats (3-month-old; 200–250 g), the Young control group. The Young control group was only kept without any intervention. Similarly, the Old control group was only kept without intervention. Except for the Young control group and the Old control group which were kept without any intervention, the other 3 groups received different interventions as follows: The Exercise group (Exe) consisted of aged rats subjected to treadmill exercise at a speed of 17 m/min. The Curcumin group (Cur) included aged rats that were given curcumin at a dosage of 50 mg/kg. The Exercise + Curcumin group (Exe + Cur) involved aged rats that received curcumin and treadmill exercise. All experiments were conducted over a period of two months [30]. The study design and timeline is presented in Fig. 1.

### 2.2 Exercise protocol

Over the course of a week, the exercise groups (Exe and Exe + Cur) became accustomed to running on treadmill (Further details can be found in Fig. 1). The animals needed to have engaged in at least one month of treadmill exercise to be included, otherwise they were excluded from the study [30]. The rats in the other groups were held in the same conditions as the exercised rats, but they were not exercising at all. All of the studies took place between 9 a.m. and 12 p.m. The training plan which is described in the timeline in Fig. 1, is regarded as mild training [31].

### 2.3 Curcumin therapy

Curcumin (Merck Chemicals Company, Darmstadt, Germany) dissolution was achieved by mixing it with a 5 % DMSO solution. To enhance solubility, we utilized the conventional method of heating the solute-solvent mixture to 70–80 °C. In addition to their regular diet, the animals were orally administered the mentioned curcumin solution at a dose of 50 mg/kg daily for 8 weeks. To ensure that the effects observed are due to curcumin and not the DMSO vehicle, and that all animals experience a similar level of stress or discomfort from the gavage procedure itself, all animal groups were gavaged with a similar dose of DMSO vehicle [32].

### 2.4 ELISA for tissue processing and protein measurement

At the end of the trial, in order to withdraw blood samples from the heart, Ketamine (80 mg/kg) and Xylazine (5 mg/kg) were administered to sedate the animals. Then, blood samples were immersed in a water bath set at 37 °C for 15 min; and then underwent a 10-min, 1000-rpm centrifugation. Protein levels were determined using serum samples, which were kept at −70 °C until TNF-α, IL-1β, IL-6, and IL-10 concentration levels were determined. Following that, the homogenate underwent centrifugation for 20 min at 1000 rpm while being kept at a temperature of 4 °C. The last stage involved removing the supernatant and extract the target proteins for ELISA analysis. The concentration levels of IL-1β (E-EL-R0012), TNF-α (RK06275), IL-6 (RK00020), and IL-10 (RK00050) were evaluated using kits for ELISA with a high level of sensitivity (ZellBio, Ulm, Germany) as outlined in the instructions given by the manufacturer.

### 2.5 Real-time polymerase chain reaction (RT-PCR) for miRNA expression analysis

The RT-PCR was utilized to determine miR-21 and miR-146a expression. Each RNA sample was subjected to three separate tests. The miRCURY™ RNA Isolation Kit (Exiqon, Vedbaek, Denmark) was used to extract miRNA from serum samples in accordance with the specified instructions from the manufacturer. Separation of RNA from other cellular components was achieved by employing a spin column as the segregation matrix, while a specialized resin served as an alternative matrix for RNA separation. Analysis of the RNA concentration and purity was conducted by employing a Nanodrop 1000 spectrophotometer (Thermo Scientific, Wilmington, DE, USA) at a wavelength of 260–280 nm. To generate the cDNA, the LNA universal RT miRNA PCR kit (Exiqon, Vedbaek, Denmark) was employed. Synthesis of cDNA involved the polyadenylation of total RNA containing miRNA, followed by the use of a poly (T) primer with a 3′ degenerate anchor and a 5′ universal tag. The amplification of cDNA was monitored using SYBR Green qPCR Mix (Exiqon, Vedbaek, Denmark). Rotor-Gene™ 6000 (Corbett Life Science, Sydney, Australia) was utilized for real-time PCR. The 2^–(ΔΔCt)^ technique was utilized to find out the relative quantitative levels of miR-21 and miR-146a, the endogenous control miRNAs.

## 3 Statistical analysis

Mean ± standard deviation (mean ± SD) was used to present the quantitative data for a sample size of seven animals in each of the five groups; and statistical analysis was performed using SPSS software version 16 (IBM, Chicago, IL, USA) to determine the significance. In order to determine group differences, one-way analysis of variance (ANOVA) was used, which was followed by Tukey's test. In this study, the threshold for statistical significance was set at a P-value below 0.05 (p < 0.05).

## 4 Results

### 4.1 MDA levels in serum

**Fig. 2:**
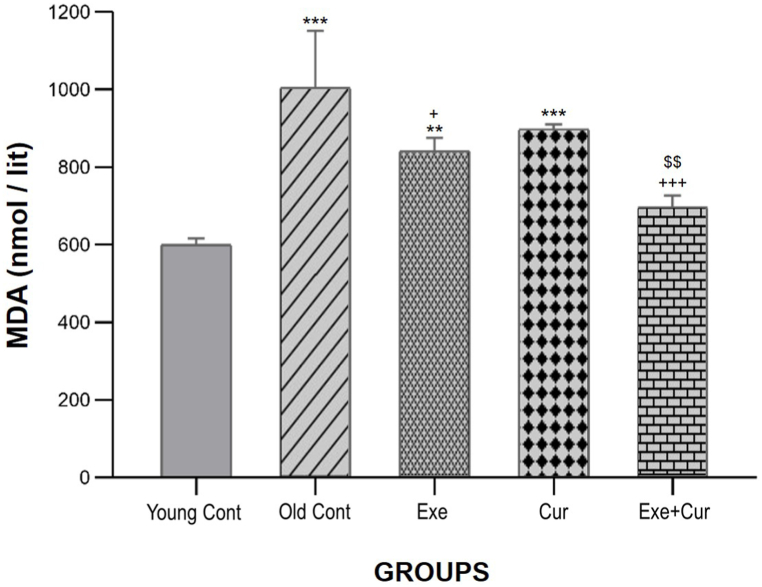
Effects of 8-week exercise and curcumin treatments on MDA in rats serum; sampled and analyzed on day 56. Bars represent the mean ± SD (n = 7); ∗∗∗p < 0.001, ∗∗p < 0.01 vs. the Young control group, ^+++^p < 0.001, ^+^p < 0.5 vs. the Old control group; ^$$^p < 0.01 vs. Curcumin group.

According to the results of the TBARS method used to analyze MDA in Fig. 2, the Old control group exhibited a significant increase in MDA levels compared to the young control group (p < 0.001). Nevertheless, following the 8-week exercise treatment, MDA levels significantly decreased (p < 0.05) when compared to the Old control group; curcumin did not have a significant effect. Notably, the Exe + Cur group displayed significantly lower MDA levels compared to the Old control (p < 0.001) and Cur (p < 0.01) groups, but not the Exe group. Remarkably, MDA levels in the Exe + Cur group were similar to those in the young control group.

### 4.2 IL-6, IL-1β, and IL-10 concentrations in serum

**Fig. 3:**
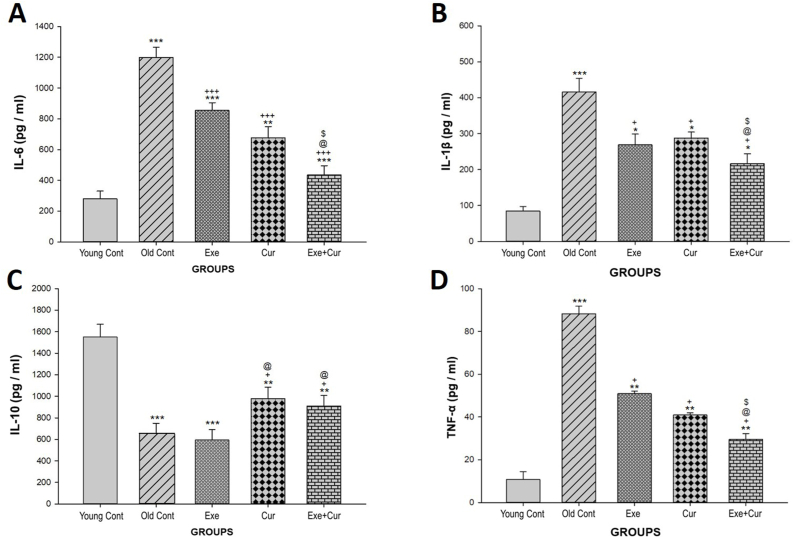
Effects of 8-week exercise and curcumin treatments on IL-6 **(A)**, IL-1β **(B)**, IL-10 **(C)**, TNF-α **(D)** levels in rats serum; sampled and analyzed on day 56. Bars represent the mean ± SD (n = 7); ∗∗∗p < 0.001, ∗∗p < 0.01, ∗p < 0.05 vs. the Young control group; ^+++^p < 0.001 vs. the Old control group; ^+^p < 0.05 vs. the Old control group; ^@^p < 0.05 vs. the Exercise group; ^$^p < 0.05 vs. Curcumin group.

Fig. 3A and B displays the ELISA results for IL-6 and IL-1β analyses. A significant increase in IL-6 (p < 0.001) and IL-1β (p < 0.001) levels in the Old control group, compared to the young control group, was observed. However, significantly lower IL-6 (p < 0.001) and IL-1β (p < 0.05) levels were observed following the 8-week exercise and curcumin treatment when compared to the Old control group. In addition, exercise and curcumin combined therapy had a decreasing synergistic effect on IL-6 (p < 0.05) and IL-1β (p < 0.05) levels in comparison to each of the exercise and curcumin groups.

In Fig. 3C, it can be observed that the serum level of IL-10 (an anti-inflammatory cytokine) was significantly reduced in the Old control group when compared to the Young control group (P < 0.001). Furthermore, the Curcumin group exhibited a significant increase in IL-10 level (P < 0.05) compared to the Old control group. Interestingly, exercise alone did not have an impact on IL-10 levels. Nevertheless, when exercise was combined with curcumin therapy, there was a significant increase in IL-10 levels (p < 0.05) in comparison to both the Exercise group and the Old control group.

### 4.3 TNF-α serum level

The results in Fig. 3D illustrate a significant increase (p < 0.001) in TNF-α serum levels within the Old control group in comparison with the Young control group. Remarkably, the application of exercise and curcumin treatment lowered TNF-α levels significantly (p < 0.05) compared to the Old control group. Meanwhile, the combined therapy exhibited a significant decrease in TNF-α serum levels, significantly diverging from both the Old control group (p < 0.05) and the Exercise group (p < 0.05).

Regarding Fig. 3A, C, and 3D, in the combination therapy, the curcumin effect on the IL-10, IL-6, and TNF-α levels was highlighted more prominently than that of exercise. With respect to Fig. 3B, it appeared that the improving effect of the combined therapy on the IL-1β level was mediated mainly by exercise.

### 4.4 Combined effect of exercise and curcumin on miR-21

**Fig. 4:**
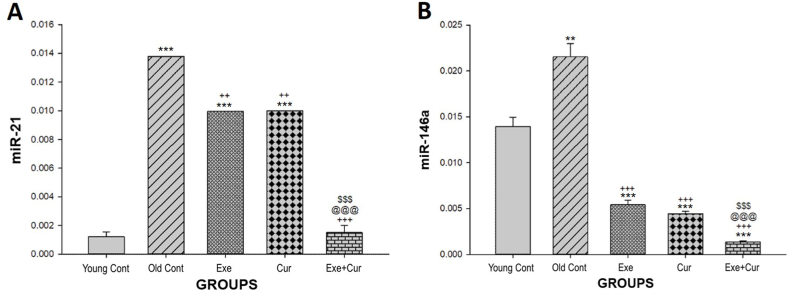
Effects of 8-week exercise and curcumin treatments on the expression of miR-21 **(A)** and miR-146a **(B)** in rats serum; sampled and analyzed on day 56. Bars represent the mean ± SD (n = 7); ∗∗∗p < 0.001, ∗∗p < 0.01 vs. the Young control group; ^+++^p < 0.001, ^++^p < 0.01 vs. the Old control group; ^@@@^p < 0.001 vs. the Exercise group; ^$$$^p < 0.001 vs. Curcumin group.

As shown in Fig. 4A, the Old control group expression of miR-21 was increased significantly compared to the Young control group (p < 0.001). However, following curcumin administration and performing exercise, the expression level of miR-21 was significantly (p < 0.01) down-regulated in the Curcumin and Exercise groups when compared to the Old control group. Moreover, miR-21 expression decreased significantly in the Exe + Cur group compared with the Old control group (p < 0.001). Additionally, a significant distinction was observed between the Exe + Cur group and the Exercise and Curcumin groups (p < 0.001) and the remarkable thing is that the Exe + Cur group was able to bring back the level of miR-21 to the same level as the Control group.

### 4.5 Combined effect of exercise and curcumin on miR-146a

In Fig. 4B, it is evident that the animals treated with curcumin (p < 0.001), exercise (p < 0.001), and a combination of curcumin and exercise (p < 0.001) exhibited significantly lower expression levels of miR-146a compared to the Old control group. Furthermore, the Exe + Cur group displayed a significant decrease in miR-146a expression compared to both the Exercise group (p < 0.001) and the Curcumin group (p < 0.001).

## 5 Discussion

Our research yielded significant results, outlined as follows: Firstly, aging was found to increase the levels of IL-1β, IL-6, TNF-α, and MDA, while reducing the level of IL-10, thus leading to increased inflammation in aged rats. However, the administration of curcumin and performing exercise led to a reduction in the levels of IL-1β, IL-6, TNF-α, MDA; and an increase in IL-10 serum level. Secondly, in aging rats, the expression levels of both miR-21 and miR-146a were observed to be elevated, contributing to inflammation. However, after an 8-week treatment of curcumin and exercise, a significant decline was recorded in the expression rates of miR-21 and miR-146a. This study aimed to investigate the potential protective effects of curcumin and exercise against age-induced inflammation in rats, as well as to uncover the underlying mechanisms involved. Advanced age is accompanied by an elevated incidence of various chronic diseases, wherein oxidative stress and inflammation play significant roles as causative factors [33]. Additionally, accumulating evidence suggests that cumulative oxidative stress and low-grade inflammation are involved in the natural aging process, independent of specific diseases [34]. Therefore, a proactive approach in the form of preventive medicine targeting the modulation of these interconnected responses appears to be the most effective strategy to address premature aging and age-related conditions including diabetes, CVD, and dementia.

Previous studies have indicated a connection between oxidative stress and chronic inflammation, with oxidative stress being closely associated with several chronic inflammatory diseases [35,36]. Still, the precise molecular pathway underlying this relationship has not been definitively established yet.

Zhang et al. have shown that aging caused an increase in pro-inflammation cytokines (IL-6, IL-1β) and in contrast, a decline of the anti-inflammatory cytokine IL-10, [37] And, according to Machi et al., the serum levels of TNF-α were found to be increased in the old groups compared to the young control group, suggesting the presence of inflammation in the older animals [38]. This increase in TNF-α may be associated with the observed impairment of cardiac and functional effects of aging in the female rats [38]. Additionally, elevated serum levels of oxidative stress of aged rats have been frequently reported [39].

Our research findings indicate a significant increase in inflammation (characterized by elevated levels of IL-6, IL-1β, and TNF-α) and oxidative stress (indicated by high levels of MDA) throughout the aging process. However, the precise mechanism underlying the modulation of the oxidative stress and inflammation loop in the development of aging remains unclear.

Recent discoveries have highlighted dysregulated miRNA expression as a distinctive feature of age-related diseases [40]. The interplay between ROS and miRNAs has been implicated in the progression of age-related diseases [41], necessitating further investigation into the nature of this relationship. Additionally, miRNAs play a crucial role in cellular senescence through post-transcriptional downregulation of target gene expression [40].

Furthermore, our research revealed an increased expression level of inflammatory miRNAs, including miR-21 and miR-146a, during senescence. Consistent with our findings, previous studies have associated these miRNAs with aging. For instance, miR-146a has been demonstrated to increase with age [42], while miR-21 is elevated in aged heart and brain tissues [43,44]. Notably, miR-21 has shown potential as a biomarker for aging, as it regulates various biological processes related to exercise adaptation, inflammation, and apoptosis [45]. The systemic levels of miR-21 have demonstrated an upregulation among elderly individuals, prompting discussions about its potential role as an inflammatory marker [46,47]. Additionally, a study conducted by Kangas et al. observed a notable increase in serum levels of miR-146a beyond the age of 69 [45].

According to another study, ROS was found to have a role in regulating the expression and function of miR-21 in highly metastatic breast cancer cell lines [43]. Furthermore, NF-κB activation by ROS led to the induction of miR-21, which played a role in arsenic-induced cell transformation [43]. According to Gong et al., miR-146a showed a positive correlation with cellular senescence in both in vitro and in vivo settings [48]. Additionally, IL-6, the cytokine implicated in the inflammatory response, may be modulated by miR-146a in the process of inflammaging, suggesting a complex interplay between miRNAs and cytokines in the progression of age-related diseases [49]. All in all, miR-146a may contribute to aging, particularly in oxidative stress-induced aging.

It has been shown that miR-21 induces expression of IL-6, TNF-α, and NF-κB from macrophages [50]. The findings indicate that the activation of miR-21 brings about a decline in the expression of PPARα, thereby diminishing the inhibitory influence of PPARα on AP-1 activation. Consequently, this mechanism promotes the upregulation of inflammation markers within the endothelium [51].

The role of miR-146a in B lymphocyte differentiation in the spleen of patients with knee osteoarthritis and inflammation induction has been widely recognized [52]. Our study, in line with previous research, indicates that elevated levels of miR-21 and miR-146a in aged animals are associated with higher levels of oxidative stress and inflammation. Consequently, it is crucial to identify novel antioxidant agents that can delay aging and mitigate age-related diseases through pharmacological intervention.

Moreover, our investigation revealed that exercise and curcumin supplementation led to decreased levels of inflammation, miR-21, and miR-146a. This finding aligns with the work of Chen et al., who reported that curcumin can reduce inflammation in cancer cell lines by suppressing miR-21 [53]. Additionally, studies have demonstrated that curcumin reduces the inflammatory response in the temporal lobe by downregulating the expression of miR-146a [54]. Supplementing with curcumin, a powerful compound known for its antioxidant and anti-inflammatory properties, can halt hepatic cellular senescence and the release of aging-associated compounds in older mice, as curcumin supplementation lowered the activity of aging-related genes in the liver, boosted antioxidant capabilities, and subdued MAPK signaling pathways. This led to better insulin balance, weight reduction, and lessening of inflammatory molecules and Senescence-Associated Secretory Phenotypes (SASPs). These findings hint that using curcumin as a supplement might be a promising approach to ward off liver cell aging [55]. Furthermore, 30 days of exercise has been shown to reduce levels of endoplasmic reticulum stress (ERS) markers, including caspase 12, caspase 8, and C/EBP Homologous Protein (CHOP) in animal models of diabetes, thereby improving liver health [56].

Furthermore, according to Porsani et al. acute exercise was found to decrease miR-21 levels in the serum of athletes, while aerobic training significantly reduced miR-146a expression in the renal tissue of male rats [57]. They also demonstrated a decrease in miR-21 following treadmill training in an experimental model of focal cerebral ischemia in rats [57]. Comparably, our study revealed that exercise reduced miR-21 and miR-146a expression in the serum of aged animals.

**Fig. 5:**
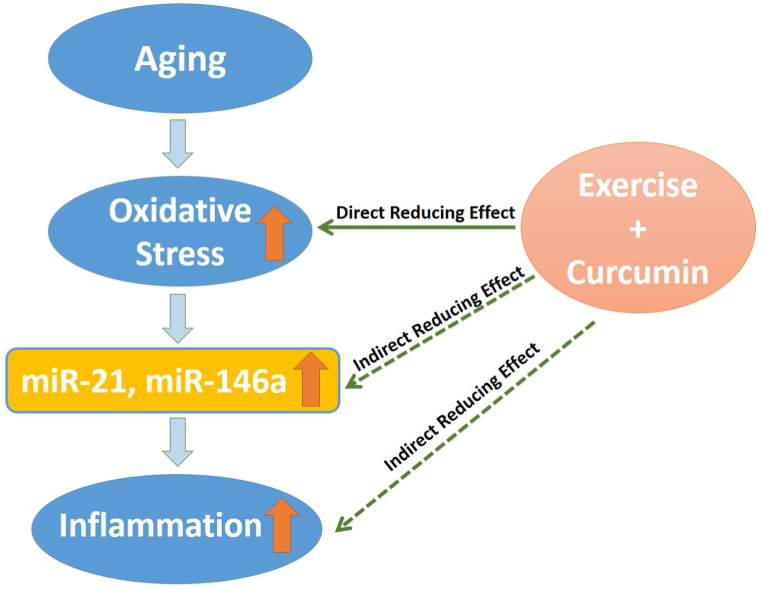
Study hypothesis. The possible relationship between oxidative stress, inflammation and microRNA is depicted.

Notably, curcumin supplementation combined with exercise exhibited a synergistic reducing effect on miR-21 and miR-146a expression, as well as on inflammation. The combined intervention also decreased MDA levels and the expression of IL-6 and TNF-α, while significantly increasing IL-10 levels. As mentioned earlier, multiple scientific investigations have revealed the ability of exercise and curcumin to reduce oxidative stress and inflammation in various organs. Accordingly, it is plausible to suggest that the age-related increase in inflammation levels may be attributed to the elevated production of miR-21 and miR-146a induced by oxidative stress. Conversely, by mitigating oxidative stress, curcumin and exercise effectively downregulate the expression of miR-21 and miR-146a, ultimately leading to a reduction in inflammation. It is likely that the combination therapy exerts more pronounced effects on intracellular signaling pathways. The main hypothesis of this study is depicted in Fig. 5. It is worth noting that utilizing anti-miR-21 and miR-146a would be beneficial in confirming this hypothesis and clarifying the roles of miRNAs, a limitation for better confirming the hypothesis and understanding the roles of miRNAs that is acknowledged in the current study. Another limitation is the limited solubility of curcumin in water, which can pose a challenge in studies that require gavage feeding to rats. Curcumin's poor solubility in water can lead to difficulties in formulating a stable and homogeneous solution for gavage feeding. This issue can impact the accuracy and consistency of dosing in animal studies, potentially affecting the reliability and reproducibility of results. Researchers may need to explore alternative solubilization methods or delivery formulations to overcome this limitation and ensure the effectiveness of curcumin administration in rat studies.

In accordance with our findings, an 8-week treatment with curcumin and exercise in old rats brought about a significant decrease in the expression of miR-21 and miR-146a. As mentioned earlier, these miRNAs directly influence oxidative stress, thereby reducing serum MDA levels and subsequently alleviating inflammation. Our results were supported by previous studies [52,54]. The impact of endurance training on the expression of miR-21 and its downstream targets in mice with breast cancer demonstrates that curcumin and exercise, either individually or in combination, can effectively reduce miR-21 and miR-146a gene expression [53].

As a final word, our study on the combined effect of exercise and curcumin on inflammation-associated microRNAs and cytokines in old male rats sheds light on one of the potential approaches for exploring the broader benefits of curcumin in old age. Future research could investigate additional mechanisms by which curcumin may positively impact aging, including mitigating cognitive impairment, metabolic dysfunction, and cellular senescence in aging organisms.

For example, research similar to studies demonstrating curcumin's role in alleviating diet-induced spatial memory impairment and hepatic metabolism in Alzheimer's disease-induced (3xTg-AD) mice [58], or its ability to reduce cellular senescence by modulating the MAPK/NF-κB signaling pathway in aged mice [55], could offer valuable insights into the diverse applications of curcumin in promoting healthy aging.

By exploring the various impacts of curcumin in aging biology, future studies may uncover novel therapeutic strategies that utilize the natural properties of this compound to enhance overall health and well-being in the elderly population.

## 6 Conclusion

In conclusion, our study brings attention to the role of miR-21 and miR-146a in oxidative stress-induced inflammation and their modulation by curcumin and exercise therapy. The findings suggest that these interventions may serve as promising strategies for reducing inflammation and mitigating age-related diseases. Further research is warranted in order to gain a deeper comprehension of the underlying mechanisms and optimize the therapeutic approach.

## CRediT authorship contribution statement

**Zahra Shargh:** Writing – original draft, Visualization, Investigation, Formal analysis, Data curation. **Keyvan Asghari:** Writing – original draft, Visualization, Investigation, Formal analysis, Data curation. **Morteza Asghariehahari:** Writing – review & editing, Visualization, Methodology, Formal analysis. **Leila Chodari:** Writing – review & editing, Writing – original draft, Visualization, Supervision, Project administration, Methodology, Investigation, Funding acquisition, Formal analysis, Data curation, Conceptualization.

## Ethics declarations

This study was reviewed and approved by the Animal Ethics Committee of Urmia University of Medical Sciences, with the approval number: [IR.UMSU.REC.1399.184].

## Additional information

No additional information is available for this paper.

## Data availability statement

The data used and analyzed in the current study are available from the corresponding author upon a reasonable request.

## Funding statement

Support of this investigation by the 10.13039/501100016286Urmia University of Medical Sciences, through a grant, is gratefully acknowledged.

## Declaration of competing interest

The authors declare that they have no known competing financial interests or personal relationships that could have appeared to influence the work reported in this paper.
